# Predicting incident fatty liver using simple cardio-metabolic risk factors at baseline

**DOI:** 10.1186/1471-230X-12-84

**Published:** 2012-07-06

**Authors:** Ki-Chul Sung, Bum-Soo Kim, Yong-Kyun Cho, Dong-il Park, Sookyoung Woo, Seonwoo Kim, Sarah H Wild, Christopher D Byrne

**Affiliations:** 1 Division of Cardiology, Department of Medicine, Kangbuk Samsung Hospital, Sungkyunkwan University School of Medicine, Seoul, Republic of Korea; 2 Division of Gastroenterology, Department of Medicine, Kangbuk Samsung Hospital, Sungkyunkwan University School of Medicine, Seoul, Republic of Korea; 3 Biostatistics Team, Samsung Biomedical Research Institute, Seoul, Republic of Korea; 4 Centre for Population Health Sciences, University of Edinburgh, IDS Building, Southampton General Hospital, MP 887, Southampton, UK; 5 Nutrition and Metabolism Unit, Faculty of Medicine, University of Southampton, IDS Building, Southampton General Hospital, MP 887, Southampton, UK; 6 NIHR Southampton Biomedical Research Centre, University Hospital Southampton, IDS Building, Southampton General Hospital, MP 887, Southampton, UK; 7 Endocrinology and Metabolism Unit, University of Southampton, IDS Building, Southampton General Hospital, MP 887, Tremona Road, Southampton, UK, SO166YD; 8 Division of Cardiology, Kangbuk Samsung, Hospital, Sungkyunkwan University School of Medicine, #108, Pyung Dong, Jongro-Ku, Seoul, 110-746, Republic of Korea

**Keywords:** Non alcoholic fatty liver disease, Fatty liver, Etiology, Risk prediction, Metabolic syndrome

## Abstract

**Background:**

Non alcoholic fatty liver disease (NAFLD) is associated with increased risk of type 2 diabetes and chronic liver disease but identifying patients who have NAFLD without resorting to expensive imaging tests is challenging. In order to help identify people for imaging investigation of the liver who are at high risk of NAFLD, our aim was to: a) identify easily measured risk factors at baseline that were independently associated with incident fatty liver at follow up, and then b) to test the diagnostic performance of thresholds of these factors at baseline, to predict or to exclude incident fatty liver at follow up.

**Methods:**

2589 people with absence of fatty liver on ultrasound examination at baseline were re-examined after a mean of 4.4 years in a Korean occupational cohort study. Multi-variable logistic regression analyses were used to identify baseline factors that were independently associated with incident fatty liver at follow up. The diagnostic performance of thresholds of these baseline factors to identify people with incident fatty liver at follow-up was assessed using receiver operating characteristic (ROC) curves.

**Results:**

430 incident cases of fatty liver were identified. Several factors were independently associated with incident fatty liver: increased triglyceride (per mmol/l increase) OR 1.378 [95%CIs 1.179, 1.611], p < 0.0001; glucose (per mmol/l increase) OR 1.215 [95%CIs 1.042, 1.416], p = 0.013; waist (per cm increase) OR 1.078 [95%CIs 1.057, 1.099], p < 0.001; ALT (per IU/L increase) OR 1.009 [95%CIs 1.002, 1.017], p = 0.016; and platelets (per 1x10^9^/L increase) OR 1.004 [1.001, 1.006], p = 0.001; were each independently associated with incident fatty liver. Binary thresholds of the five factors were applied and the area under the ROC curve for incident fatty liver was 0.75 (95%CI 0.72–0.78) for the combination of all five factors above these thresholds.

**Conclusion:**

Simple risk factors that overlap considerably with risk factors for type 2 diabetes allow identification of people at high risk of incident fatty liver at who use of hepatic imaging could be targeted.

## Background

Prevalence estimates for non alcoholic fatty liver disease (NAFLD) range from 17% to 33% in general populations in Western countries [1,2] and it is predicted that NAFLD will soon become the most important contribution to demand for liver transplantation [3]. NAFLD is also associated with type 2 diabetes, cardiovascular disease [4,5] and insulin resistance [6] in muscle, adipose and liver [7].

Patients with possible NAFLD are treated by a range of specialists [8] who have to decide which patients are at sufficiently high risk to investigate further to make the diagnosis. Readily available single anthropometric tests or blood tests lack sufficient sensitivity or specificity to identify patients with NAFLD. For example, a body mass index (BMI) ≥25 kg/m^2^ as a measure of overweight or obesity, is not specific for NAFLD, because not all overweight or obese people have fatty liver; and an alanine transaminase (ALT) value > 31U/l in men and >19 IU/l in women is fairly sensitive but is not a specific test for NAFLD [9]. It is therefore difficult to select appropriate patients for expensive radiological diagnostic imaging tests such as ultrasound, because single tests lack sufficient sensitivity and specificity to identify high risk patients. Although other more sensitive techniques than ultrasound are available for detecting liver fat, e.g. proton magnetic resonance spectroscopy (MRS) [10,11], MRS is an expensive diagnostic test and is also imperfect at identifying the extent or severity of NAFLD. Consequently, ultrasound is usually the preferred initial radiological test employed by most clinicians to establish the presence of moderate or large amounts of liver fat.

There is increasing interest in developing algorithms or risk scores for identifying liver fat, because accumulation of liver fat or some component of fat metabolism is the prerequisite for progression of NAFLD to steatohepatitis and fibrosis [12]. Several investigators have undertaken cross sectional studies examining relationships between risk factors and prevalent fatty liver [13-17], but the ability of these risk factors, or algorithms, to predict incident fatty liver in a prospective study is uncertain.

Recently, in a cross sectional study of people with and without diabetes, and people recruited from gastroenterology clinics, Kotronen et al. developed a NAFLD liver fat score based on serum insulin, aspartate transaminase (AST), ALT concentrations, presence or absence of metabolic syndrome and type 2 diabetes. These investigators validated the fat score against the presence and quantity of hepatic fat as assessed by MRS [18]. In this study, the optimum liver fat score cut-point had 86% sensitivity and 71% specific for predicting NAFLD [18]. Although these data suggest an excellent performance of the liver fat score, an accompanying editorial suggested the score was ‘not ready for prime time’ [19], not least because of the cross sectional study design and the limited potential applicability of these findings to general populations as some participants were recruited from hospital clinics.

We have undertaken a cohort study of people who were shown to be free from hepatic steatosis on ultrasound examination at baseline, and who had a further ultrasound examination at follow up an average of 4.4 years later. We used these data to identify individuals who had developed new (incident) fatty liver during the follow up period. Since there are no guidelines to help identify which patients should undergo ultrasound testing to diagnose fatty liver, the aim of our study was to: a) identify easily measured risk factors at baseline that were independently associated with incident fatty liver at follow up, and then b) to test the diagnostic performance of thresholds of these factors to predict or to exclude incident fatty liver at follow up.

## Methods

**Figure 1:**
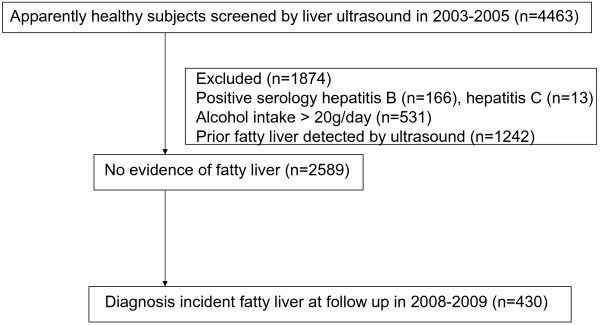
Flow chart summarizing the selection of study participants.

Electronic medical records were used to identify 4463 participants from a cohort of Korean employees who had had an occupational health check in 2003–2005 that included collection of data on anthropometry and liver ultrasound. Information on smoking history (never, current, or past) and whether individuals participated in any regular exercise was obtained by questionnaire. A follow up examination was performed in 2008 and 2009 at Kangbuk Samsung Hospital in Seoul, Korea and people without fatty liver at baseline were only included if they had a further ultrasound at follow-up (Figure 1). The institutional review board of Kangbuk Samsung Hospital approved this study.

We excluded participants with positive serologic marker for hepatitis B (n = 188) or hepatitis C virus (n = 13) and alcohol consumption in excess of 20 g/day (n = 531). Regular exercise and smoking status were assessed by a physician administered questionnaire. Individuals were asked if they had participated in any regular exercise at least once a week and smoking status was classified as never, ex and current smoker. Data were specifically requested from the central source on those individuals who had a complete data-set for all these measurements.

Plasma and serum were collected after at least 12 h of fasting. Glucose was measured using the hexokinase method (Advia 1650 Autoanalyzer; Bayer Diagnostics, Leverkusen, Germany). Insulin was measured with an immunoradiometric assay (Biosource, Nivelle, Belgium) (intra- and interassay coefficient of variation of 2.1–4.5% and 4.7–12.2%, respectively). An enzymatic calorimetric test was used to measure total cholesterol and triglyceride concentrations. High density lipoprotein cholesterol (HDLc) concentration was measured by the selective inhibition method and a homogeneous enzymatic calorimetric test was used to measure low density lipoprotein cholesterol (LDLc) concentration (Advia 1650 Autoanalyzer). High-sensitivity C-reactive protein (hs-CRP) was analysed by performing particle-enhanced immunonephelomety using the BN System (Dade Behring, Marburg, Germany). HOMA-IR (Homeostasis Model Assessment of Insulin Resistance) was calculated as a measure of insulin resistance [20]. Abdominal ultrasonography (Logic Q700 MR; GE, Milwaukee,WI, USA) was performed in all subjects by experienced clinical radiologists and fatty liver was diagnosed based on known standard criteria, including hepatorenal echo contrast, liver brightness, and vascular blurring, using a 3.5 MHz probe [21].

### Risk prediction modeling for fatty liver comparing established and population-specific newly optimized thresholds

All the observations including ultrasound and blood tests that are included in the analyses were undertaken at base-line and data from a further ultrasound performed at follow up was available for people without fatty liver at baseline. Established thresholds for metabolic risk factors (waist, glucose and triglyceride) were identified according to the Joint Interim Statement 2009 criteria for the metabolic syndrome (MetS) [22].

For ALT, we used sex specific cut-offs for normal liver function that have been shown to exclude NAFLD [9]. For platelet number, we used the lower limit of normal for Kangbuk Samsung Hospital, Korea as the cut point (158 × 10^9^/l).

Additionally, we also identified new population-specific optimized thresholds of each variable that showed the highest accuracy (based on the area under receiver operating curves, AUROC) for identifying people at high risk of incident fatty liver, and the best accuracy (NPV%) for excluding incident fatty liver at follow up; using the Youden index method [23].

### Validation of the performance of established thresholds and population-specific thresholds in training and validation data-sets

We established training and validation data-sets to validate the performance of the established and new population-specific thresholds. To develop training and validation data-sets, we split the data for each stratum into training and validation data-sets, using random sampling methodology to ensure similar distribution of sex and body mass index (BMI) between both data sub-sets.

### Statistical analysis

The descriptive statistics for continuous variables are presented using means and standard deviations (SDs) for normally distributed variables and medians and ranges for non-normally distributed variables. Categorical variables were described using frequencies (percentages). Continuous variables were compared between two groups using *t*-test or Mann–Whitney U tests and categorical variables were analyzed using chi-squared test or Fisher’s exact test. To identify baseline factors that were independently associated with incident fatty liver at follow up, logistic regression analysis was undertaken, and the odds ratio (OR) and 95% confidence intervals for incident fatty liver were calculated for continuous variables. The diagnostic performance of binary criteria for baseline biochemical and anthropometric tests that were independently associated with incident fatty liver at follow up was assessed using ROC curves using both established cut-points used to define the MetS and cut-points identified within the study population. All statistical analyses were performed using SPSS 17.0 (SPSS Inc., Chicago, IL, USA) and Stata 11.0 (StataCorp LP, 4905 Lakeway Drive College Station, Texas 77845 USA).

## Results

We identified 988 people with prevalent fatty liver and 2589 people who did not have ultrasound evidence of fatty liver, positive hepatitis B or C serology or excessive alcohol consumption at baseline examination. Of these 2589 people, 430 developed incident fatty liver, during the mean follow-up period of average 4.37 years (1596 days). Table 1 shows a comparison of baseline characteristics (a) between individuals with and without fatty liver at baseline and (b) between individuals with and without incident fatty liver at follow up. Mean HOMA-IR was higher in the group with fatty liver compared with the group without fatty liver at baseline (2.69 ± 1.16 v. 1.95 ± 0.69, p ≤ 0.0001). There were also a higher percentage of people with MetS in the group with fatty liver compared with the group without fatty liver at baseline (29.7% v. 5.2%, p ≤ 0.0001). A higher proportion of men (23.4%) than women (9.7%) developed incident fatty liver (p < 0.0001). At baseline, there was a higher HOMA-IR, and a higher prevalence of MetS, in the people that developed fatty liver during the follow up period (n = 430) compared with those people who remained free from developing fatty liver (n = 2159) (Table 1).

**Table 1:** Comparison of baseline characteristics (a) between individuals with and without fatty liver at baseline and (b) between individuals with and without incident fatty liver at follow up

	**Baseline fatty liver status (a)**			**Follow up fatty liver status (b)**		
	**Absent**	**Present**	**(a)**	**Absent**	**Present**	**(b)**
			**p value**			**p value**
n	2589	988		2159	430	
Age (y)	46.27 ± 8.51	48.08 ± 8.69	<0.0001	46.06 ± 8.57	47.32 ± 8.11	0.005
% Men	1305(50.4%)	800(81.0%)	<0.0001	999 (46.3%)	306 (71.2%)	<0.0001
BMI (kg/m^2^)	22.94 ± 2.58	26.10 ± 2.42	<0.0001	22.66 ± 2.53	24.33 ± 2.32	<0.0001
Waist	77.00 ± 8.44	87.52 ± 6.68	<0.0001	75.93 ± 8.25	82.36 ± 7.22	<0.0001
Exercise						
None	817(31.5%)	270(27.3%)	.002	689 (31.9%)	128 (29.8%)	0.81
≤1time/week	359(13.9%)	183(18.5%)		297 (13.8%)	62 (14.4%)	
>1time/week	1413(54.6%)	535(54.1%)		1173 (54.3%)	240 (55.8%)	
% Smoker						
Never	1821(70.3%)	490(49.6%)	<0.0001	1581 (73.2%)	240 (55.8%)	<0.0001
Past	293(11.3%)	238(24.1%)		215 (10.0%)	78 (18.1%)	
Current	475(18.3%)	260(26.3%)		363 (16.8%)	112 (26.0%)	
Alcohol (gm/day)	5.81 ± 7.07	7.68 ± 7.53	<0.0001	5.37 ± 6.82	8.01 ± 7.84	<0.0001
SBP (mmHg)	115.23 ± 13.82	112.39 ± 15.00	<0.0001	114.60 ± 13.49	118.36 ± 14.97	<0.0001
DBP (mmHg)	74.64 ± 9.89	80.08 ± 10.08	<0.0001	74.06 ± 9.71	77.53 ± 10.25	<0.0001
Triglyceride (mmol/L) Median [IQR]	1.10 [0.80,1.51]	1.80 [1.33,2.44]	<0.0001	1.04 [0.77,1.41]	1.38 [1.04,1.91]	<0.0001
HDL-C (mmol/L)	1.54 ± 0.30	1.40 ± 0.23	<0.0001	1.56 ± 0.31	1.47 ± 0.27	<0.0001
HsCRP (mg/L)	0.82 ± 1.43	1.38 ± 1.75	<0.0001	0.77 ± 1.40	1.03 ± 1.58	<0.0001
Platelets (10^9^/L)	246.14 ± 53.10	235.32 ± 50.57	<0.0001	244.73 ± 52.63	253.16 ± 54.90	0.003
Albumin (g/L)	45.6 ± 2.4	46.7 ± 2.5	<0.0001	45.4 ± 3.0	45.9 ± .2.3	<0.0001
ALT (U/L)	21.44 ± 15.19	38.39 ± 22.02	<0.0001	20.38 ± 11.67	26.69 ± 25.95	<0.0001
AST (U/L)	22.14 ± 7.11	27.99 ± 10.87	<0.0001	21.87 ± 6.93	23.46 ± 7.83	<0.0001
AST/ALT ratio	1.17 ± 0.36	0.82 ± 0.27	<0.0001	1.19 ± 0.36	1.03 ± 0.33	<0.0001
AST/platelet index	9.46 ± 3.97	11.62 ± 5.81	<0.0001	9.38 ± 3.74	9.86 ± 4.95	0.06
Fasting Glucose (mmol/L)	5.10 ± 0.66	5.55 ± 1.13	<0.0001	5.06 ± 0.56	5.26 ± 1.01	<0.0001
Fasting Insulin (pmol/L)	59.48 ± 18.65	75.39 ± 27.89	<0.0001	58.91 ± 18.18	62.28 ± 20.64	0.002
HOMA-IR	1.95 ± 0.69	2.69 ± 1.16	<0.0001	1.92 ± 0.66	2.10 ± 0.81	<0.0001
MetS, n (%)	135 (5.2%)	293 (29.7%)	<0.0001	92 (4.3%)	43 (10.0%)	<0.0001

Data are presented as mean (SD), except where indicated.

As several individual cardio-metabolic risk factors are common to insulin resistance, MetS and type 2 diabetes, we investigated the relationship between individual cardio-metabolic risk factors at baseline and incident fatty liver at follow up. The results of analyses in which all potential baseline cardio-metabolic risk factors were entered into a logistic regression model with incident fatty liver as the outcome are shown in Table 2. Age, sex, hsCRP, blood pressure, HDLc, and smoking did not have statistically significant independent associations with incident fatty liver in the fully adjusted model. In contrast, glucose, waist circumference, triglyceride concentration, alanine transaminase (ALT) and platelet number were all independently associated with incident fatty liver in this model.

**Table 2:** Odds ratios derived from multivariable logistic regression for the association between age, sex and cardio-metabolic risk factors at baseline and incident fatty liver at follow-up

	**Incident fatty at follow up**	
	**OR [95%CIs]**	**p value**
Age (per year)	0.990 [0.977, 1.004]	0.176
Male sex	1.103 [0.795, 1.531]	0.557
Glucose (per mmol/l increase)	1.215 [1.042, 1.416]	0.013
Insulin (per pmol/L increase)	1.002 [0.996, 1.008]	0.436
hsCRP (per mg/l increase)	1.286 [0.632, 2.615]	0.488
Triglyceride (per mmol/l increase)	1.378 [1.179, 1.611]	<0.0001
HDLc (per mmol/l increase)	0.821 [0.545,1.236]	0.345
ALT (per IU/L increase)	1.009 [1.002, 1.017]	0.016
Platelets (per 1x10^9^/L increase)	1.004 [1.001, 1.006]	0.001
Waist (per cm increase)	1.078 [1.057, 1.099]	<0.0001
Smoking (current vs past/never	0.923 [0.696, 1.225]	0.579
DBP (per mmHg increase)	1.003 [0.990, 1.015]	0.656

*or SBP OR [95%CIs] = 1.002[0.993, 1.011], p = 0.65.

Model adjusted for all of above variables.

The significant variables identified from regression analysis (shown in Table 2) were selected for ROC curve analyses. The performance of these variables in risk prediction modeling for incident fatty liver is summarized in Table 3. The sensitivity of established thresholds (defined in methods) of the individual risk factors to identify a group at high risk of incident fatty liver are shown in Table 3. These thresholds were very good at identifying people at low risk of incident fatty liver (negative predictive values (NPVs) for all variables were between 78.9% and 92.2%).

**Table 3:** Comparison of the performance of key variables at baseline to identify incident fatty liver at follow up, using established and newly optimized thresholds of each variable*

**Established thresholds**						
**Test**	**Cut point**	**AUROC (95%CI)**	**Sensitivity (%) (95%CI)**	**Specificity (%)(95%CI)**	**PPV (%) (95%CI)**	**NPV (%) (95%CI)**
Glucose	5.6 (mmol/l)	0.56	24.9%	86.6%	26.4%	85.7%
		(0.53–0.59)	(19.2%–31.3%)	(84.5%–88.6%)	(20.4%–33.1%)	(83.5%–87.7%)
Waist	90 (cms) men		18.4%	88.5%	31.8%	78.9%
		0.56	(12.5%–25.6%)	(85.4%–91.2%)	(22.1%–42.8%)	(75.3%–82.2%)
	80 (cms) women	(0.53–0.58)	29%	90.5%	24.7%	92.2%
			(18.2%–41.9%)	(87.8%–92.8%)	(15.3%–36.1%)	(89.7%–94.3%)
Triglyceride	1.7 (mmol/l)	0.60	35.9%	85.1%	31.6%	87.3%
		(0.57–0.64)	(29.4%–42.8%)	(82.8%–87.1%)	(25.8%–38.0%)	(85.2%–89.3%)
ALT	30 (IU/l)		32.7%	75.7%	28.1%	79.5%
	(men)	0.52	(25.2%–40.9%)	(71.7%–79.4%)	(21.5%–35.4%)	(75.6%–83%)
	19 (IU/l)	(0.49–0.56)	32.3%	69.4%	10.2%	90.5%
	(women)		(20.9%–45.3%)	(65.4%–73.1%)	(6.31%–15.2%)	(87.4%–93.1%)
Platelets	158 (x10^9^/l)	0.51	97.6%	3.32%	16.3%	87.8%
		(0.49–0.52)	(94.5%–99.2%)	(2.34%–4.57%)	(14.3%–18.5%)	(73.8%–95.9%)
**Population–specific thresholds**						
**Test**	**Cut point**	**AUROC (95%CI)**	**Sensitivity (%) (95%CI)**	**Specificity (%) (95%CI)**	**PPV (%) (95%CI)**	**NPV (%) (95%CI)**
Glucose	5.0 mmol/l	0.58	70.8%	45.4%	20.0%	89.0%
		(0.55–0.62)	(64.1%–76.9%)	(42.4%–48.4%)	(17.2%–23.1%)	(86.1%–91.5%)
Waist	78.8 cms (men)		87.7%	35.2%	28.2%	90.8%
		0.68	(81.0%–92.7%)	(30.9%–39.6%)	(24.1%–32.5%)	(85.8%–94.4%)
	73.5 cms (women	(0.65–0.71)	72.4%	67.9%	19.1%	95.6%
			(59.1%–83.3%)	(63.8%–71.8%)	(14.3%–24.8%)	(93.2%–97.4%)
Triglyceride	1.1 mmol/l	0.64	76.1%	52.2%	23.5%	91.9%
		(0.61–0.67)	(69.7%–81.7%)	(49.2%–55.2%)	(20.3%–26.9%)	(89.4%–93.9%)
ALT	24 (IU/l)		55.8%	60.7%	29.2%	82.5%
	(men)	0.54	(47.4%–64%)	(56.3%–65.0%)	(23.9%–34.9%)	(78.3%–86.2%)
	13 (IU/l)	(0.51–0.57)	85.5%	27.5%	11.2%	94.6%
	(women)		(74.2%–93.1%)	(23.9%–31.3%)	(8.53%–14.4%)	(90.1%–97.5%)
Platelets	262 (x10^9^/l)	0.56	47.4%	64.9%	20.7%	86.5%
		(0.52–0.60)	(40.4%–54.4%)	(62.0%–67.8%)	(17.1%–24.6%)	(83.9%–88.8%)

Since there has been recent debate as to whether there should be population-specific thresholds for waist circumference as a key feature of the metabolic syndrome, we tested the diagnostic performance of population-specific thresholds that showed the best discrimination for each of the five factors (waist circumference, glucose, triglyceride, ALT and platelets), to identify or exclude incident fatty liver. The results of applying the newly defined population-specific thresholds of each of these variables are also shown in Table 3. These data demonstrated that, for this population, lower thresholds of glucose (5.0 mmol.l^−1^), waist circumference (78.8 cms (men) and 73.6 cms (women)), triglyceride (1.1 mmol.l^−1^), ALT (24 IU. l^−1^ (men), and 13 IU. .l^−1^ (women)) and a higher platelet count (262x10^−9^.l^−1^) than the established or laboratory based thresholds), resulted in slightly better diagnostic performance of each test to identify a high risk group for fatty liver. These population-specific thresholds also improved performance of each test to exclude fatty liver (with the exception of ALT in men).

We examined the diagnostic performance of combinations of the variables (waist circumference, and concentrations of glucose, triglyceride and ALT, and platelet count) to identify high risk of incident fatty liver, using both the established, and the population-specific measurement thresholds. When all five risk factors (waist circumference, and concentrations of glucose, triglyceride and ALT, and platelet count) were combined, for established thresholds, the area under receiver operator curve (AUROC) value for incident fatty liver was 0.65 (95%CI 0.61, 0.69), with a positive predictive value (PPV) of 50.0% (95%CI 15.7, 84.3) and an NPV of 84.0% (95%CI 81.9, 86.0); whereas for the population-specific thresholds, the AUROC for incident fatty liver was 0.75 (95%CI 0.72, 0.78), with a PPV of 40.0% (95%CI 15.7, 84.3) and an NPV of 85.4% (95%CI 83.3, 87.3). We used the population-specific criteria to calculate the number and proportion of people who did not have fatty liver at baseline who met all five thresholds or who met the ALT threshold alone as these criteria are most likely to be applied in clinical practice to inform a decision to request a liver ultrasound examination. The number (%) of people who met all five specific criteria was 87 (3.4%) whereas 585 people (23%) met the ALT threshold alone. Consequently, 585 people might be selected for an ultrasound examination, based upon the presence of an ALT concentration above the population-specific threshold for ALT, whereas in contrast only 87 people would be selected for ultrasound investigation, by using the presence of all five criteria as the essential pre-requisite.

Next we compared the diagnostic performance of combining the five factors in the training and the validation datasets. The performance of both sets of thresholds was very similar in the training and validation data-sets (Table 4).

**Table 4:** Performance (AUROC and 95%CIs) of combination of baseline tests for identifying incident fatty liver in training data-set and validation data-set

	**training data-set**	**validation data-set**
**New thresholds**		
(A) glucose, waist, triglyceride, ALT, platelets	0.75	0.71
	(95% CI : 0.72–0.78)	(95% CI : 0.67–0.74)
**Established thresholds**		
(B) glucose, waist, triglyceride, ALT, platelets	0.65	0.68
	(95% CI : 0.61–0.69)	(95% CI : 0.65–0.72)

A) Combining thresholds of glucose, waist, triglyceride, ALT and platelets using new optimized measurement thresholds, and

(B) Combining thresholds of glucose, waist, triglyceride, ALT and platelets using established measurement thresholds.

## Discussion

Our study is the first to combine identification of risk factors with the subsequent testing of these factors in risk prediction modeling for incident fatty liver at follow up. Whilst others have examined relationships between risk scores (or cardio-metabolic risk factors) and prevalent fatty liver in cross sectional studies [13-17], we have examined the AUROC, NPV and PPV of risk factors identified at baseline, to predict incident cases of fatty liver at average follow up of 4.4 years. We show that combination of thresholds of five easily measured risk factors (waist circumference, glucose, ALT, platelets and triglyceride) allows identification of the small proportion of people at high risk of incident fatty liver who could then be selected for hepatic imaging tests. Such an approach would focus the use of relatively expensive non-invasive diagnostic imaging tests, such as ultrasound, and would be likely to increase the specificity of this imaging test to diagnose fatty liver, and lead to more cost effective use of limited resources. Additionally, our data suggest that in this cohort the population-specific thresholds are possibly better at diagnosing, or identifying, people at high risk of fatty liver (than the established thresholds). In contrast, the established criteria are possibly better at identifying people at low risk of, or excluding, fatty liver.

Interestingly, the factors that we have identified as being associated with increased risk of developing fatty liver are also strongly associated with risk of type 2 diabetes either directly, (glucose, triglyceride, ALT and waist circumference) [24], or as a consequence of their association with the metabolic syndrome (platelet count) [25]. This finding clearly emphasizes the importance of shared risk factors for both cardio-metabolic and fatty liver diseases in a general middle-aged population and strongly suggests a common etio-pathogenesis for type 2 diabetes and NAFLD. Although age is a strong risk factor for type 2 diabetes, age was not an independent risk factor for NAFLD in this occupational cohort, perhaps because the age range was fairly narrow and because the effect of age is at least partly mediated through other factors included in our model.

The unexpected finding that platelet count was associated with increased risk of fatty liver may be explained by the fact that platelets are a potential mediator of inflammation [26,27] and some of the individuals with incident fatty liver at follow up may have developed a more severe form of NAFLD with inflammation and fibrosis (non alcoholic steatohepatitis). We suggest that research is needed to explore further the relationship between platelet activation and development of NAFLD. Recent findings that both platelet-derived serotonin and platelet release of the key profibrogenic mediators CXC Chemokine Ligand 4 and transforming growth factor-β (TGF-β), all support the notion that platelet activation may be involved in NAFLD progression [27,28].

The diagnostic performance of ultrasound, computed tomography (CT), T1-weighted dual-echo magnetic resonance (MR) imaging, and point-resolved proton (hydrogen 1[(1)H]) MR spectroscopy in the assessment of hepatic steatosis has previously been compared. MR spectroscopic measurements of hepatic fat and ultrasound measurement of liver fat both correlate well with histopathologic steatosis assessment [29]. Furthermore, compared with histopathologic steatosis assessment (as the gold standard), the sensitivity of ultrasound and MR spectroscopy, was 65% and 91%, respectively, whereas the specificity was 77% and 87% respectively. Thus, in establishing an initial diagnosis of NAFLD it is accepted that ultrasound has an acceptable balance of sensitivity and specificity to detect liver fat, particularly where there is >30% liver tissue fat infiltration [30]. Although ultrasonography is a reasonably accurate technique for detecting modest amounts of liver fat (>30% liver fat infiltration), ultrasound has poor sensitivity to detect minor amounts of fatty infiltration [30] and it would be important to test the ability of our five simple cardio-metabolic risk factors to predict lesser amounts of liver fat. However, it was not possible for us to undertake this analysis in our prospective study design as an MRS investigation at baseline and at follow up would have been required in 2589 people.

There are several strengths to our study. There is a considerable body of published research that has investigated cross sectional associations of risk factors with liver fat. To our knowledge, no previous studies have described the etiological factors associated with incident NAFLD in a longitudinal study, and then tested the utility of these factors in risk prediction modeling in a general population. Other studies have used more sensitive tests for detecting liver fat, such as the MRS-based study that focused on developing a risk score (for liver fat) [18]; but these investigators employed cross sectional analyses, in a population that was not solely recruited from a general population. Moreover the investigators used measurements of insulin concentrations that are also not readily available to the primary care physician. Recently, using abdominal ultrasonography, Tsuneto et al. [31] examined 1635 Nagasaki atomic bomb survivors (606 men) biennially without fatty liver at baseline, for a mean follow-up of 11.6+/−4.6 years. In all, 323 (124 men) incident fatty liver cases were diagnosed. The incidence of fatty liver was 19.9/1000 person-years (22.3 for men, 18.6 for women) and peaked in the sixth decade of life. In multivariate analysis, obesity (RR, 2.55; P < 0.001), hypertriglyceridemia (RR, 1.92; P < 0.001) and hypertension (RR, 1.31; P = 0.046) were independently associated with incident fatty liver. Our results are largely in agreement with these findings, and our work on risk prediction extends this work. Tsuneto et al. showed a borderline significant independent association between blood pressure and fatty liver, a finding that was not confirmed in our study. Interestingly, despite the younger age distribution, the incidence rate for fatty liver was higher in our study (at 34.7/1000 person years).

Our study has some limitations. We have used routine clinical data so that although ultrasonography was undertaken by experienced clinical radiologists, a formal assessment of inter-observer variability was not possible. As the incidence rate for NAFLD is probably highest in the sixth decade [31], and because our study population included a relatively narrow age range with a preponderance of men, our estimates of fatty liver incidence cannot be extrapolated to the whole population. We do not have data describing behaviour during the follow up period, and therefore we are unable to comment as to whether there was any change between baseline and follow up measurements that may have affected risk of fatty liver. As ultrasound does not detect liver fat ≤30% fat infiltration, it is possible that some people had small amounts of liver fat present at baseline, which was not detectable. We have studied a single population largely comprising one ethnic group and consequently, our results cannot be extrapolated to other ethnic groups. However, our cohort comprises an ethnic group who are typical of a large percentage of the population of Northeast Asia that represents as many as 1.5 billion people. We were unable to exclude subjects taking drugs known to be associated with increased risk of fatty liver as these data were not available, although in this population-based cohort, this is likely to be a very small percentage of the total cohort.

## Conclusions

We have identified simple baseline etiological factors (waist circumference, glucose, triglyceride and ALT concentrations, and platelet number) that are associated with incident fatty liver over a follow up of an average of 4.4 years. Importantly, we have shown that the diagnostic performance of either the population-specific or the established thresholds used in the definition of the MetS is such that either could be used in clinical practice to focus limited resources and identify a high risk subgroup for fatty liver. We suggest that this selected sub-group should receive a liver ultrasound and may derive particular benefit from lifestyle change and assessment and management of diabetes risk as well as the hepatic disease risk. We suggest that thresholds of these five factors combined, could be used as a screening tool to select people who are particularly likely to, either already have, or to develop fatty liver. Such an approach would improve the use and specificity of ultrasound imaging in a more targeted approach to the diagnosis of fatty liver.

## Abbreviations

NAFLD: nonalcoholic fatty liver disease; MetS: metabolic syndrome; ALT: alanine aminotransaminases; AST: aspartate transaminase; ROC: receiver operator characteristic curves; AUC: area under curve; AUROC: area under receiver operator characteristic curve; PPV: positive predictive value; NPV: negative predictive value; hs-CRP: high-sensitivity C-reactive protein; HDLc: high density lipoprotein cholesterol; SBP: systolic blood pressure; DBP: diastolic blood pressure; TG: triglyceride; BMI: body mass index.

## Competing interests

The authors declare that they have no competing interests.

## Author contributions

K-CS and CDB designed the study and wrote the manuscript. SHW contributed to study design and to writing the manuscript. B-SK, Y-KC and D-I P contributed to the manuscript. SW and SK analysed the data. All authors read and approved the final manuscript.

## Pre-publication history

The pre-publication history for this paper can be accessed here:

http://www.biomedcentral.com/1471-230X/12/84/prepub

## References

[B1] Ruhl CE Everhart JE Determinants of the association of overweight with elevated serum alanine aminotransferase activity in the United States Gastroenterology 2003 124 71 79 10.1053/gast.2003.50004 12512031

[B2] Clark JM Diehl AM Defining nonalcoholic fatty liver disease: Implications for epidemiologic studies Gastroenterology 2003 124 248 250 10.1053/gast.2003.50032 12512048

[B3] Schreuder TC Verwer BJ van Nieuwkerk CM Mulder CJ Nonalcoholic fatty liver disease: an overview of current insights in pathogenesis, diagnosis and treatment World J Gastroenterol 2008 14 2474 2486 10.3748/wjg.14.2474 18442193 PMC2708357

[B4] Neuschwander-Tetri BA Caldwell SH Nonalcoholic steatohepatitis: summary of an AASLD Single Topic Conference Hepatology 2003 37 1202 1219 10.1053/jhep.2003.50193 12717402

[B5] Targher G Bertolini L Rodella S Tessari R Zenari L Lippi G Arcaro G Nonalcoholic fatty liver disease is independently associated with an increased incidence of cardiovascular events in type 2 diabetic patients Diabetes Care 2007 30 2119 2121 10.2337/dc07-0349 17519430

[B6] Marchesini G Bugianesi E Forlani G Cerrelli F Lenzi M Manini R Natale S Vanni E Villanova N Melchionda N Rizzetto M Nonalcoholic fatty liver, steatohepatitis, and the metabolic syndrome Hepatology 2003 37 917 923 10.1053/jhep.2003.50161 12668987

[B7] Holt HB Wild SH Wood PJ Zhang J Darekar AA Dewbury K Poole RB Holt RI Phillips DI Byrne CD Non-esterified fatty acid concentrations are independently associated with hepatic steatosis in obese subjects Diabetologia 2006 49 141 148 16323001 10.1007/s00125-005-0070-x

[B8] Vuppalanchi R Chalasani N Nonalcoholic fatty liver disease and nonalcoholic steatohepatitis: Selected practical issues in their evaluation and management Hepatology 2009 49 306 317 10.1002/hep.22603 19065650 PMC2766096

[B9] Prati D Taioli E Zanella A Della TE Butelli S Del VE Vianello L Zanuso F Mozzi F Milani S Conte D Colombo M Sirchia G Updated definitions of healthy ranges for serum alanine aminotransferase levels Ann Intern Med 2002 137 1 10 12093239 10.7326/0003-4819-137-1-200207020-00006

[B10] Szczepaniak LS Babcock EE Schick F Dobbins RL Garg A Burns DK McGarry JD Stein DT Measurement of intracellular triglyceride stores by H spectroscopy: validation in vivo Am J Physiol 1999 276 E977 E989 10329993 10.1152/ajpendo.1999.276.5.E977

[B11] Cassidy FH Yokoo T Aganovic L Hanna RF Bydder M Middleton MS Hamilton G Chavez AD Schwimmer JB Sirlin CB Fatty liver disease: MR imaging techniques for the detection and quantification of liver steatosis Radiographics 2009 29 231 260 10.1148/rg.291075123 19168847

[B12] Byrne CD Olufadi R Bruce KD Cagampang FR Ahmed MH Metabolic disturbances in non-alcoholic fatty liver disease Clin Sci (Lond) 2009 116 539 564 10.1042/CS20080253 19243311

[B13] Handberg A Hojlund K Gastaldelli A Flyvbjerg A Dekker JM Petrie J Piatti P Beck-Nielsen H Plasma sCD36 is associated with markers of atherosclerosis, insulin resistance and fatty liver in a nondiabetic healthy population J Intern Med 2012 271 294 304 10.1111/j.1365-2796.2011.02442.x 21883535

[B14] Gastaldelli A Kozakova M Hojlund K Flyvbjerg A Favuzzi A Mitrakou A Balkau B Fatty liver is associated with insulin resistance, risk of coronary heart disease, and early atherosclerosis in a large European population Hepatology 2009 49 1537 1544 10.1002/hep.22845 19291789

[B15] Wang PW Hsieh CJ Psang LC Cheng YF Liou CW Weng SW Chen JF Chen IY Li RH Eng HL Fatty liver and chronic inflammation in Chinese adults Diabetes Res Clin Pract 2008 81 202 208 10.1016/j.diabres.2008.04.014 18534708

[B16] Bedogni G Kahn HS Bellentani S Tiribelli C A simple index of lipid overaccumulation is a good marker of liver steatosis BMC Gastroenterol 2010 10 98 10.1186/1471-230X-10-98 20738844 PMC2940930

[B17] Bedogni G Bellentani S Miglioli L Masutti F Passalacqua M Castiglione A Tiribelli C The Fatty Liver Index: a simple and accurate predictor of hepatic steatosis in the general population BMC Gastroenterol 2006 6 33 10.1186/1471-230X-6-33 17081293 PMC1636651

[B18] Kotronen A Peltonen M Hakkarainen A Sevastianova K Bergholm R Johansson LM Lundbom N Rissanen A Ridderstrale M Groop L Orho-Melander M Yki-Jarvinen H Prediction of non-alcoholic fatty liver disease and liver fat using metabolic and genetic factors Gastroenterology 2009 137 865 872 10.1053/j.gastro.2009.06.005 19524579

[B19] Chalasani N Nonalcoholic fatty liver disease liver fat score and fat equation to predict and quantitate hepatic steatosis: promising but not prime time! Gastroenterology 2009 137 772 775 10.1053/j.gastro.2009.07.032 19638269

[B20] Matthews DR Hosker JP Rudenski AS Naylor BA Treacher DF Turner RC Homeostasis model assessment: insulin resistance and beta-cell function from fasting plasma glucose and insulin concentrations in man Diabetologia 1985 28 412 419 10.1007/BF00280883 3899825

[B21] Saverymuttu SH Joseph AE Maxwell JD Ultrasound scanning in the detection of hepatic fibrosis and steatosis Br Med J (Clin Res Ed) 1986 292 13 15 10.1136/bmj.292.6512.13 PMC1338970 3080046

[B22] Alberti KG Eckel RH Grundy SM Zimmet PZ Cleeman JI Donato KA Fruchart JC James WP Loria CM Smith SC Harmonizing the metabolic syndrome: a joint interim statement of the International Diabetes Federation Task Force on Epidemiology and Prevention; National Heart, Lung, and Blood Institute; American Heart Association; World Heart Federation; International Atherosclerosis Society; and international association for the Study of Obesity Circulation 2009 120 1640 1645 10.1161/CIRCULATIONAHA.109.192644 19805654

[B23] Youden WJ Index for rating diagnostic tests Cancer 1950 3 32 35 10.1002/1097-0142(1950)3:1<32::AID-CNCR2820030106>3.0.CO;2-3 15405679

[B24] Wang J Stancakova A Kuusisto J Laakso M Identification of undiagnosed type 2 diabetic individuals by the finnish diabetes risk score and biochemical and genetic markers: a population-based study of 7232 Finnish men J Clin Endocrinol Metab 2010 95 3858 3862 10.1210/jc.2010-0012 20463095

[B25] Jesri A Okonofua EC Egan BM Platelet and white blood cell counts are elevated in patients with the metabolic syndrome J Clin Hypertens (Greenwich) 2005 7 705 711 10.1111/j.1524-6175.2005.04809.x 16330892 PMC8109429

[B26] Ripoche J Blood platelets and inflammation: Their relationship with liver and digestive diseases Clin Res Hepatol Gastroenterol 2011 35 353 357 10.1016/j.clinre.2011.02.012 21482218

[B27] Semple JW Italiano JE Freedman J Platelets and the immune continuum Nat Rev Immunol 2011 11 264 274 10.1038/nri2956 21436837

[B28] Adams LA Biomarkers of liver fibrosis J Gastroenterol Hepatol 2011 26 802 809 10.1111/j.1440-1746.2010.06612.x 21198831

[B29] van Werven JR Marsman HA Nederveen AJ Smits NJ ten Kate FJ van Gulik TM Stoker J Assessment of hepatic steatosis in patients undergoing liver resection: comparison of US, CT, T1-weighted dual-echo MR imaging, and point-resolved 1 H MR spectroscopy Radiology 2010 256 159 168 10.1148/radiol.10091790 20574093

[B30] Palmentieri B de Sio I La Mura V Masarone M Vecchione R Bruno S Torella R Persico M The role of bright liver echo pattern on ultrasound B-mode examination in the diagnosis of liver steatosis Dig Liver Dis 2006 38 485 489 10.1016/j.dld.2006.03.021 16716779

[B31] Tsuneto A Hida A Sera N Imaizumi M Ichimaru S Nakashima E Seto S Maemura K Akahoshi M Fatty liver incidence and predictive variables Hypertens Res 2010 33 638 643 10.1038/hr.2010.45 20379184

